# Circular RNA circ-DONSON facilitates gastric cancer growth and invasion via NURF complex dependent activation of transcription factor SOX4 

**DOI:** 10.1186/s12943-019-1006-2

**Published:** 2019-03-28

**Authors:** Lixian Ding, Yuying Zhao, Shuwei Dang, Yue Wang, Xinglong Li, Xiaotong Yu, Zhongsheng Li, Jiufeng Wei, Ming Liu, Guodong Li

**Affiliations:** 1 Department of General Surgery, the Fourth Affiliated Hospital of Harbin Medical University, No. 37 Yiyuan Street, Nangang District, Harbin, 150001 People’s Republic of China; 2 Bio-Bank of Department of General Surgery, the Fourth Affiliated Hospital of Harbin Medical University, Harbin, 150001 People’s Republic of China; 3 Department of Medical Oncology, The Fourth Affiliated Hospital of Harbin Medical University, Harbin, 150001 People’s Republic of China; 4 Department of Pharmacology and Toxicology, Wright State University, Fairborn, OH 45435 USA

**Keywords:** circ-DONSON, Gastric cancer, NURF, SOX4, Progression

## Abstract

**Background:**

Circular RNAs (circRNAs) are a novel type of noncoding RNAs and play important roles in tumorigenesis, including gastric cancer (GC). However, the functions of most circRNAs remain poorly understood. In our study, we aimed to investigate the functions of a new circRNA circ-DONSON in GC progression.

**Methods:**

The expression of circ-DONSON in gastric cancer tissues and adjacent normal tissues was analyzed by bioinformatics method, qRT-PCR, Northern blotting and in situ hybridization (ISH). The effects of circ-DONSON on GC cell proliferation, apoptosis, migration and invasion were measured by using CCK8, colony formation, EdU, immunofluorescence (IF), FACS and Transwell assays. qRT-PCR and Western blotting were utilized to validate how circ-DONSON regulates SOX4 expression. ChIP, DNA fluorescence in situ hybridization (DNA-FISH) and DNA accessibility assays were used to investigate how circ-DONSON regulates SOX4 transcription. The interaction between circ-DONSON and NURF complex was evaluated by mass spectrum, RNA immunoprecipitation (RIP), pulldown and EMSA assays. Xenograft mouse model was used to analyze the effect of circ-DONSON on GC growth in vivo.

**Results:**

Elevated expression of circ-DONSON was observed in GC tissues and positively associated with advanced TNM stage and unfavorable prognosis. Silencing of circ-DONSON significantly suppressed the proliferation, migration and invasion of GC cells while promoting apoptosis. circ-DONSON was localized in the nucleus, recruited the NURF complex to SOX4 promoter and initiated its transcription. Silencing of the NURF complex subunit SNF2L, BPTF or RBBP4 similarly attenuated GC cell growth and increased apoptosis. circ-DONSON knockdown inhibited GC growth in vivo.

**Conclusion:**

circ-DONSON promotes GC progression through recruiting the NURF complex to initiate SOX4 expression.

**Electronic supplementary material:**

The online version of this article (10.1186/s12943-019-1006-2) contains supplementary material, which is available to authorized users.

## Background

Gastric cancer (GC) is one of the most common cancers in gastrointestinal malignancies and remains the third leading cause of cancer-associated deaths around the world [1]. The development of GC is induced by several factors such as smoking [2], constant salty food intake [3] and genetic mutation [4]. As the developing of novel strategies for GC diagnosis and treatment, its incidence and mortality rates have been steadily decreased in the recent years [5]. However, the five-year overall survival rate of GC patients remains still lower than 29% because of tumor invasiveness and recurrence [6]. Hence, it is urgently required to investigate the molecular mechanisms of GC progression and develop more effective therapeutic methods.

Circular RNAs (circRNAs) are a recently discovered member of the noncoding RNA family and characterized by a covalently closed continuous loop and resistance to RNase R digestion [7, 8]. CircRNAs are highly stable and exist in various cell types. Increasing RNA-sequencing analyses have shown that circRNAs are highly expressed in tumor tissues, including GC tissues [9]. Emerging studies have indicated that circRNAs participate in tumorigenesis through regulating various biological processes, including proliferation, survival, invasion and differentiation [10–12]. For example, circPVT1 contributes to non-small cell lung cancer (NSCLC) cell growth and migration by inhibiting miR-125b to activate E2F2 expression [13]. Circ_0005230 is overexpressed in breast cancer and promotes tumor cell division and invasiveness through miR-618/CBX8 signaling [14]. CircMMP9 is upregulated by EIF4A3 in glioblastoma and contributes to tumor development by sponging miR-124 [15]. In GC, circ-SFMBT2 was found to initiate tumor growth [10]. These evidences demonstrate essential functions of circRNAs in cancer. However, there are still a large number of circRNAs in GC, whose roles are ill studied.

circ-DONSON (circbase ID: hsa_circ_0004339), derived from back-splicing of DONSON mRNA (from exon 3 to exon 8), is located on chromosome 21q22.11 and has 948 nucleotides in length. To our knowledge, the function of circ-DONSON has not been researched. In this study, we found that circ-DONSON was highly expressed in GC tissues and positively correlated with TNM stage and poor prognosis. circ-DONSON silencing suppressed GC cell proliferation, migration and invasion while promoting apoptosis in vitro. Moreover, circ-DONSON knockdown suppressed GC growth in vivo. Mechanistically, we demonstrated that circ-DONSON recruits the NURF complex to SOX4 promoter and initiates its transcription. In summary, circ-DONSON works as an oncogene in GC and might be a potential therapeutic target.

## Methods

### Human samples

A total of 142 GC tissues and paired adjacent normal tissues were obtained from the Fourth Affiliated Hospital of Harbin Medical University. The tissues were immediately stored in liquid nitrogen after surgery. These patients did not receive radiotherapy or chemotherapy prior to collection. This study was approved by the Ethics Committee of the Fourth Affiliated Hospital of Harbin Medical University. The informed consent was achieved from every patient.

### Cell culture

GC cell lines (BGC-823, AGS, MGC-803, MKN74, HGC-27 and SGC-7901) and normal human gastric epithelial cell line GES-1 were bought from Shanghai Institutes for Biological Sciences, China. These cells were cultured with Dulbecco’s Modified Eagle medium (DMEM) medium supplemented with 10% fetal bovine serum (FBS), 100 U/ml of penicillin, and 100 μg/ml of streptomycin (HyClone).

### Lentivirus production and cell transfection

The lentivirus-containing short hairpin RNA (shRNA) targeting circ-DONSON, BPTF, SNF2L or RBBP4 was purchased from GenePharma (Shanghai, China). Both shRNAs were transfected into the GC cell lines using Lipofectamine 3000 (Invitrogen) according to the manufacturer’s instructions. At 48 h post-transfection, the cells were selected with puromycin (2 μg/mL) for 2 weeks to construct stable cell lines. The transfection efficiency was verified by qRT-PCR.

### Quantitative real-time PCR (qRT-PCR)

Total RNAs were isolated using TRIzol and inversely transcribed into cDNA using M-MLV and the SYBR Green Master Mix kit (Takara, Otsu, Japan). qPCR was completed as described previously [16].

### Cell proliferation

Cell proliferation was determined using the Cell Counting Kit-8 (CCK8) assay according to a previous study [9].

### Colony formation assay

BGC-823 and AGS cells were planted into the 6-well plates and incubated for 12 days at 37 °C. Then colonies were fixed and stained with 0.1% crystal violet. The colony numbers were counted finally.

### 5-Ethynyl-20-deoxyuridine (EdU) incorporation assay

The EdU assay was performed using a Cell-Light EdU DNA Cell Proliferation Kit (RiboBio, Shanghai, PR, China) according to the manufacturer’s instructions.

### Transwell assay

Transwell assays were used to detect cell migration and invasion and conducted as previously described [5].

### RNA fluorescence in situ hybridization (RNA-FISH)

RNA-FISH was performed according to a previous study [5]. Briefly, the probes targeting the back-splicing site of circ-DONSON were used for this assay. The probe of circ-DONSON was marked with DIG-UTP (Roche, 11,209,256,910) for RNA labeling. Cells were first fixed with 4% paraformaldehyde for 10 min and then permeabilized in PBS with 0.5% Triton X-100 for 5 min. Next, the cells were hybridized with labeled FISH probe at 37 °C overnight. Afterwards, the cells were washed with 4× sodium citrate buffer containing 0.1% Tween-20 for 5 min and then washed with 1× SSC for 5 min. Finally, cells were stained with 4,6-diamidino-2-phenylindole for 10 min. The images were acquired using a fluorescence microscopy (Leica, SP8 laser confocal microscopy).

### Animal assay

Four-week-old BABL/c female nude mice were purchased and maintained under specific pathogen-free conditions. For the in vivo tumor formation assay, BGC-823 cells (circ-DONSON silencing or control) were injected into the right flank of BABL/c nude mice (4 mice for each group). The tumor volume was measured every 5 days. 30 days after injection, the animals were sacrificed and the xenograft tumors were dissected and weighed. All animal studies were approved by the Animal Care and Use Committee of the Fourth Affiliated Hospital of Harbin Medical University.

### Statistical analysis

Statistical analyses were carried out by using SPSS 20.0 (IBM, SPSS, Chicago, IL, USA) and GraphPad Prism. Student’s t-test or ANOVA was used to assess the statistical significance for comparisons of two groups. The Pearson’s correlation coefficient analysis was used to analyze the correlations. Overall survival (OS) and disease-free survival curves were analyzed using the Kaplane-Meier method and log-rank test. *P* < 0.05 was considered statistically significant.

## Results

### circ-DONSON is upregulated in GC tissues and positively correlated with poor prognosis

**Fig. 1:**
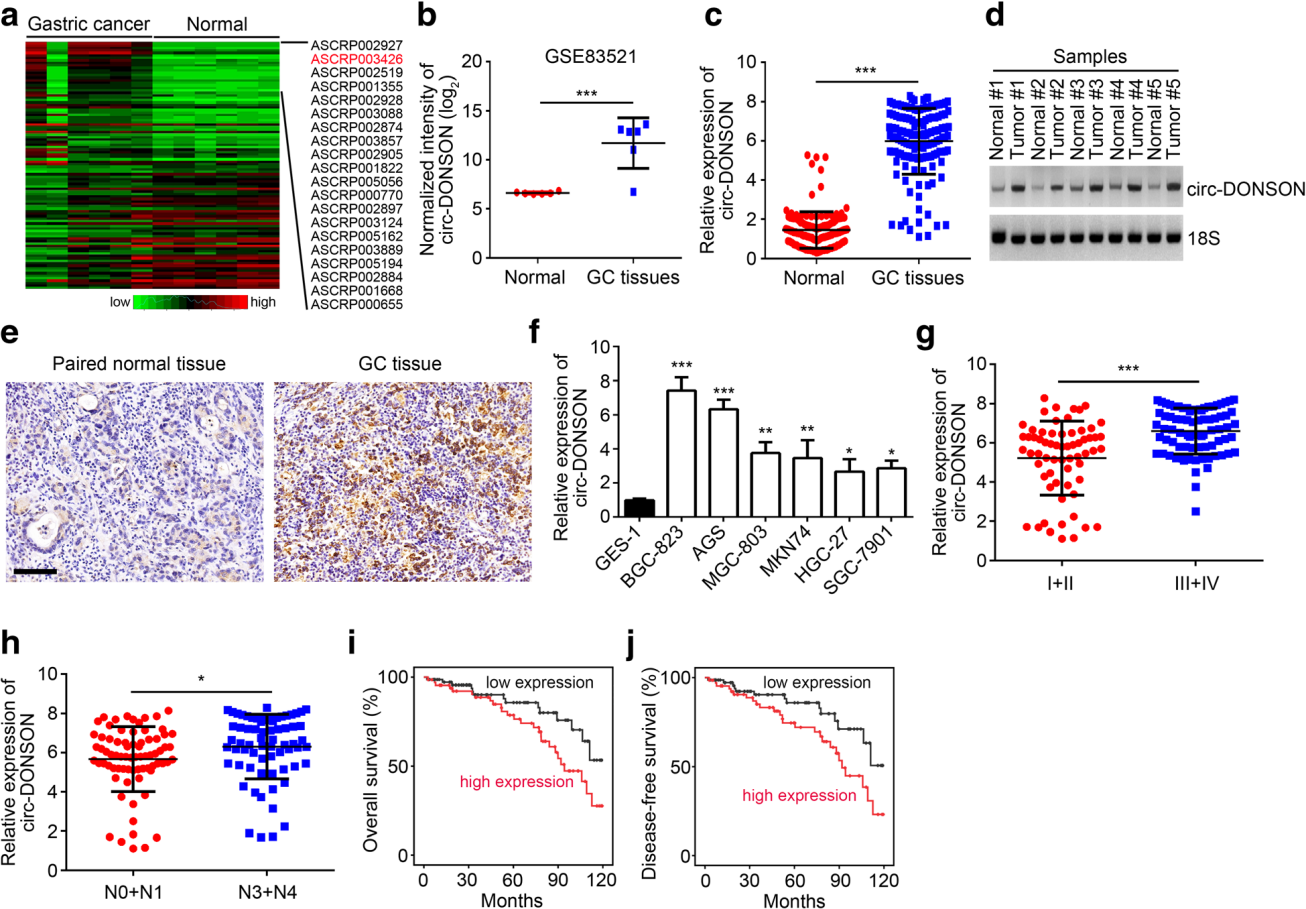
circ-DONSON is upregulated in GC tissues and positively correlated with poor prognosis. **a** Heatmap according to differentially expressed circRNAs between GC tissues and adjacent normal tissues in GSE83521 dataset. **b** Expression intensity of circ-DONSON in GC tissues and adjacent normal tissues based on GSE83521 dataset. **c** qRT-PCR analysis of relative circ-DONSON expression levels in 142GC tissues and their adjacent normal tissues. **d** Northern blotting analysis of circ-DONSON expression in GC tissues and paired normal tissues. **e** In situ hybridization (ISH) was used to analyze circ-DONSON expression in GC tissues and paired normal tissues. Scale bar: 50 μm. **f** Increased expression of circ-DONSON was observed in GC cell lines compared to GES-1 cells. **g** Relative expression of circ-DONSON in GC with different TNM stage. **h** Expression levels of circ-DONSON in GC patients with or without lymph node metastasis. **i**, **j** Kaplan-Meier plots of the overall survival and disease-free survival of GC patients with high (*n* = 68) and low (*n* = 74) levels of circ-DONSON. *P < 0.05, **P < 0.01 and ***P < 0.001

To identify important circRNAs involved in GC progression, we first analyzed overexpressed circRNAs in GC tissues compared to adjacent normal tissues according to online dataset (GSE83521). As shown, the circRNA circ-DONSON (probe ID: ASCRP003426) is the most upregulated circRNAs among all candidates (Fig. 1a, b). circ-DONSON derives from back-splicing of DONSON mRNA (Additional file 1: Figure S1a). And its sequence is presented in Additional file 1: Table S1. Then qRT-PCR analysis was conducted to validate its expression. circ-DONSON expression was significantly upregulated in 142 GC tissues compared to their adjacent normal tissues (Fig. 1c). We then measured circ-DONSON expression through Northern blotting in 5 pairs of GC tissues and adjacent normal tissues. The results indicated that circ-DONSON levels were higher in tumor tissues (Fig. 1d), which was further confirmed by in situ hybridization (ISH) (Fig. 1e). Similarly, the expression of circ-DONSON was increased in GC cell lines compared to GES-1 cell line (Fig. 1f). Then we analyzed the correlation between circ-DONSON expression and clinical features. We found that circ-DONSON expression was positively correlated with TNM stage and lymphoid metastasis (Fig. 1g, h). Furthermore, higher expression of circ-DONSON in GC patients was correlated with lower overall survival rate and disease-free survival rate (Fig. 1i, j), indicating circ-DONSON might be a prognostic marker.

### circ-DONSON silencing suppresses GC cell proliferation, migration and invasion, and induces apoptosis

**Fig. 2:**
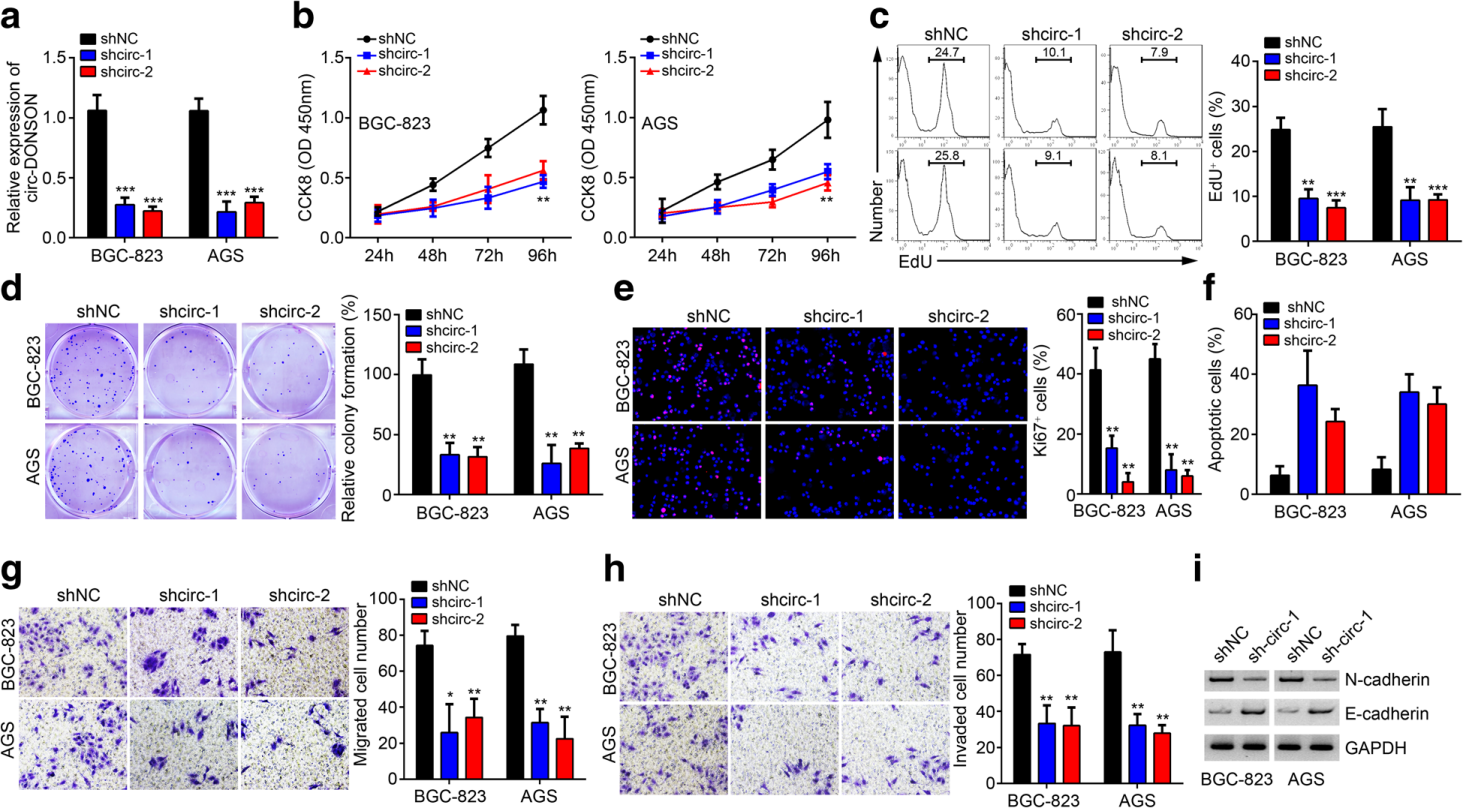
circ-DONSON silencing suppresses GC cell proliferation, migration and invasion, and induces apoptosis. **a** qRT-PCR was performed to confirm the relative expression of circ-DONSON in BGC-823 and AGS cells transfected with two independent shRNAs targeting circ-DONSON. **b**-**e** CCK8, EdU, colony formation and Ki67 staining assays was used to analyze proliferation of BGC-823 and AGS cells after circ-DONSON silencing. **f** Annexin/PI staining followed by FACS analysis indicated that circ-DONSON knockdown induced apoptosis. **g**, **h** Transwell assay illustrated that circ-DONSON knockdown suppressed the migration and invasion of BGC-823 and AGS cells. **i** Western blotting analysis of N-cadherin and E-cadherin expression in BGC-823 and AGS cells after circ-DONSON depletion. *P < 0.05, **P < 0.01 and ***P < 0.001

We next investigated the roles of circ-DONSON in GC cell phenotypes. Because circ-DONSON level was relatively higher in BGC-823 and AGS cells (Fig. 1f), we performed following experiments using these two cells. Using two independent shRNAs targeting circ-DONSON, we effectively decreased its expression in BGC-823 and AGS cells (Fig. 2a). Through CCK8 assay, we found that circ-DONSON silencing significantly inhibited the proliferation of BGC-823 and AGS cells (Fig. 2b). EdU assay also illustrated that circ-DONSON knockdown reduced the incorporation of EdU (Fig. 2c). To further confirm it, we conducted colony formation assay, and found that the colony numbers were decreased after circ-DONSON silencing (Fig. 2d). Importantly, the Ki67 positive BGC-823 and AGS cells were reduced after circ-DONSON knockdown (Fig. 2e), supporting that circ-DONSON knockdown inhibited GC cell proliferation. Then we analyzed apoptosis, migration and invasion. Results demonstrated that circ-DONSON silencing induced more apoptosis while impairing the abilities of migration and invasion (Fig. 2f-h). Interestingly, the western blotting assay showed that loss of circ-DONSON increased the epithelial marker E-cadherin expression and decreased the mesenchymal marker N-cadherin expression in BGC-823 and AGS cells (Fig. 2i). To further rule out the effect of shRNA off-target, we overexpressed circ-DONSON by transfection with pcDNA3-circ-DONSON vector in BGC-823 and AGS cells (Additional file 1: Figure S1b). CCK8 and colony formation assays indicated that circ-DONSON overexpression promoted the proliferation of BGC-823 and AGS cells (Additional file 1: Figure S1c, d). Furthermore, ectopic expression of circ-DONSON enhanced the migration and invasion of BGC-823 and AGS cells (Additional file 1: Figure S1e, f). Thus, these results demonstrated that circ-DONSON promotes GC growth and metastasis in vitro.

### circ-DONSON regulates GC cell malignant behaviors through activating SOX4

**Fig. 3:**
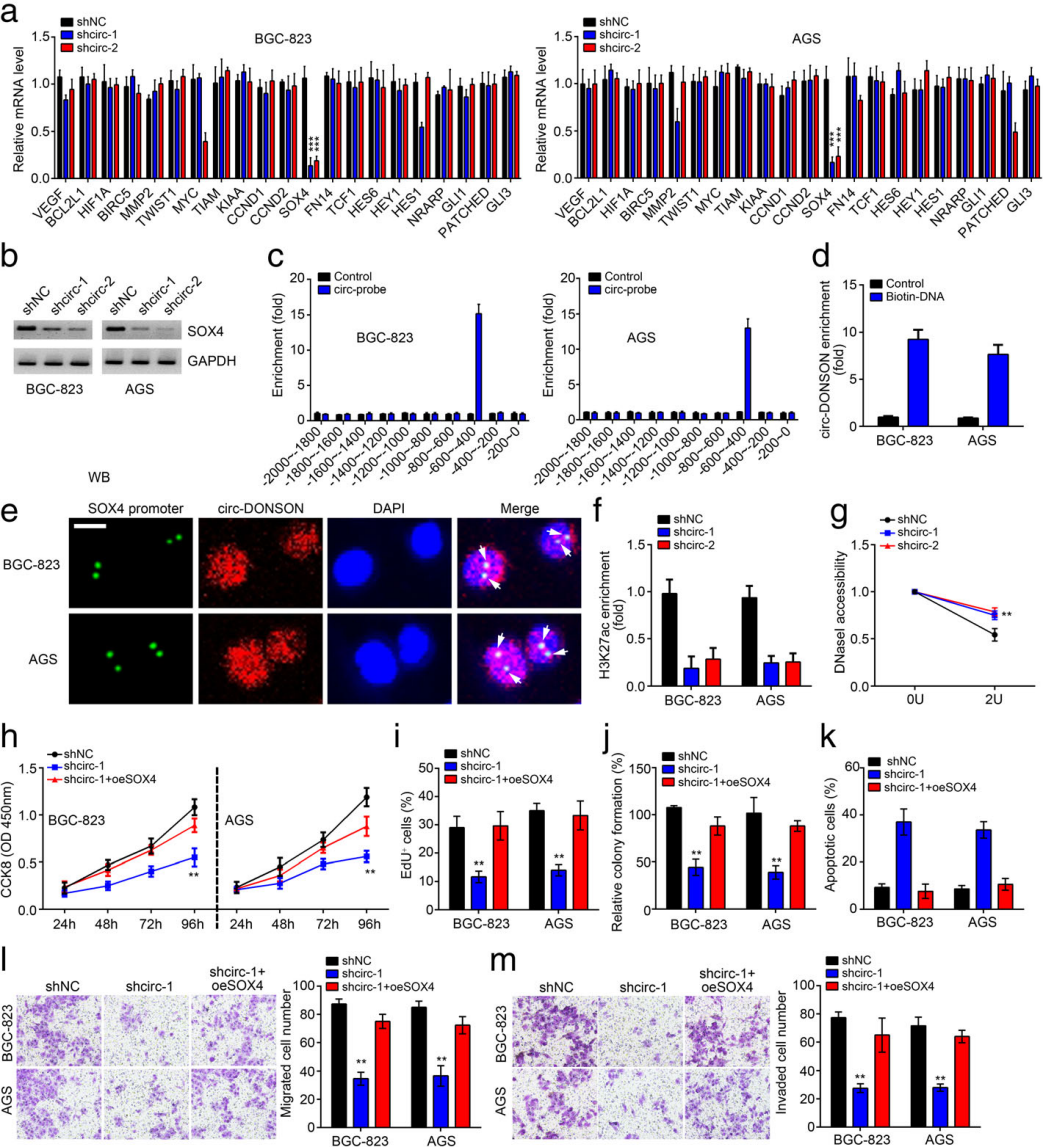
circ-DONSON regulates GC cell malignant behaviors through activating SOX4. **a** qRT-PCR analysis of indicated gene expression in BGC-823 and AGS cells after circ-DONSON depletion. **b** Western blotting result showed that circ-DONSON silencing suppressed SOX4 expression in BGC-823 and AGS cells. **c** ChIP assay was performed to measure the association of circ-DONSON with SOX4 promoter. **d** Pulldown assay showed that biotin-labeled SOX4 promoter region precipitated circ-DONSON in BGC-823 and AGS cell lysates. **e** DNA-FISH assay indicated the co-localization between circ-DONSON and SOX4 promoter in BGC-823 and AGS cells. Scale bar: 5 μm. **f** ChIP assay showed that circ-DONSON silencing led to decreased enrichment of active marker H3K27ac on SOX4 promoter in BGC-823 and AGS cells. **g** SOX4 promoter was more resistant to DNaseI digestion after circ-DONSON knockdown. **h**-**j** CCK8, EdU and colony formation assays were performed to detect cell proliferation. **k** Restoration of SOX4 reduced the apoptosis of BGC-823 and AGS cells induced by circ-DONSON silencing. **l**, **m** Restoration of SOX4 rescued the abilities of migration and invasion in circ-DONSON knocked down BGC-823 and AGS cells. **P < 0.01 and ***P < 0.001

Previous studies have reported that key signaling pathways, such as NOTCH signaling, Wnt signaling, NF-κB signaling and Hedgehog signaling, play crucial roles in tumorigenesis [17, 18]. Thus, we speculated whether circ-DONSON regulates these signaling pathways. We measured the effects of circ-DONSON knockdown on the activation of these pathways by evaluating the expression of their target genes (HES6, HEY1, HES1, NRARP for NOTCH signaling; MYC, TIAM, KIAA, CCND1, CCND2, SOX4, FN14, TCF1 for Wnt signaling; VEGF, BCL2L1, HIF1A, BIRC5, MMP2, TWIST1 for NF-κB signaling; GLI1, PATCHED, GLI3 for Hedgehog signaling). Interestingly, we found that circ-DONSON silencing only significantly led to decreased expression of SOX4 (Fig. 3a, b), implying circ-DONSON might directly regulate SOX4 expression. Using circ-DONSON specific probes, we found that circ-DONSON could enrich on SOX4 promoter region (− 600 ~ − 400 bp from the transcription start site) (Fig. 3c). Pulldown assay also indicated that biotin labeled SOX4 promoter DNA precipitated circ-DONSON in cell lysates (Fig. 3d). Consistently, FISH assay showed that SOX4 promoter was co-localized with circ-DONSON in BGC-823 and AGS cells (Fig. 3e), suggesting circ-DONSON might regulate SOX4 transcription. As shown, we really found that circ-DONSON silencing suppressed the enrichment of transcriptional active marker H3K27ac on SOX4 promoter (Fig. 3f). Moreover, after circ-DONSON knockdown, the SOX4 promoter was more resistant to DNaseI digestion (Fig. 3g), indicating circ-DONSON regulates SOX4 promoter accessibility. To further demonstrate that whether circ-DONSON-mediated SOX4 transcription promotes GC progression, we overexpressed SOX4. Through CCK8, EdU and colony formation assays, we found that SOX4 overexpression enhanced the proliferation of circ-DONSON-depleted BGC-823 and AGS cells (Fig. 3h-j). Moreover, restoration of SOX4 also reversed the effects of circ-DONSON silencing on apoptosis, migration and invasion (Fig. 3k-m). Taken together, circ-DONSON activates SOX4 transcription to promote GC progression.

### circ-DONSON associates with the NURF complex by directly interacting with SNF2L subunit

**Fig. 4:**
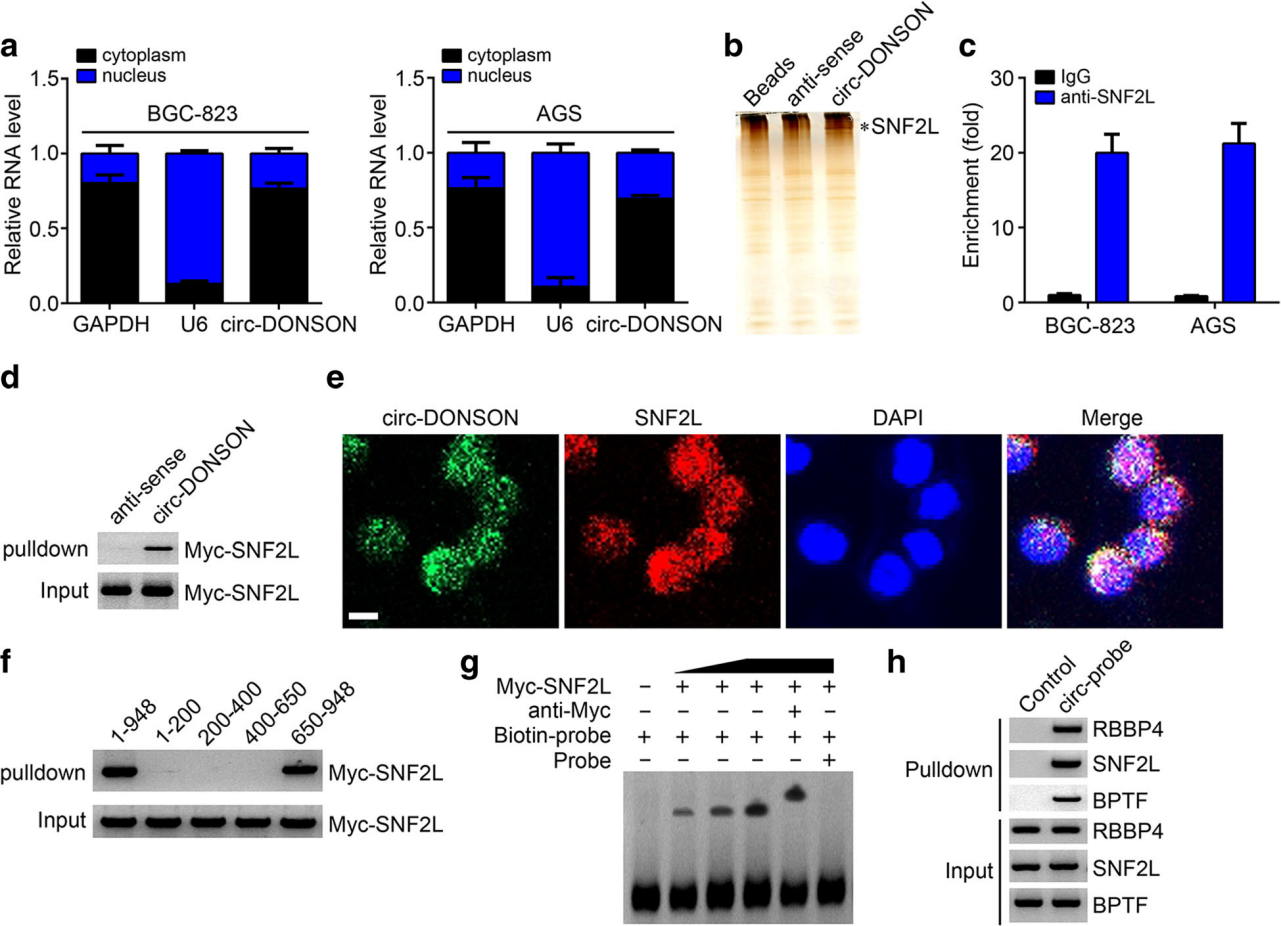
circ-DONSON associates with the NURF complex by directly interacting with SNF2L subunit. **a** qRT-PCR analysis showed that circ-DONSON was mainly localized in the nucleus of GC cells. **b** biotin-labeled linear circ-DONSON was used for incubation with BGC-823 cell lysates, followed by silver staining and mass spectrum identification. SNF2L was identified as a candidate for interaction with circ-DONSON. **c** RIP assay using anti-SNF2L showed that SNF2L precipitated circ-DONSON in BGC-823 and AGS cell lysates. **d** Pulldown assay confirmed that biotin-labeled linear circ-DONSON interacted with MYC-SNF2L. **e** RNA-FISH assay verified the colocalization between circ-DONSON and SNF2L in BGC-823 cells. Scale bar: 5 μm. **f** Domain mapping assay indicated that the region of 650–948 bp in circ-DONSON was essential for the interaction with SNF2L. **g** RNA-EMSA assay confirmed the interaction of circ-DONSON (650–948 bp) with SNF2L. **h** Pulldown assay using probes targeting circ-DONSON indicated that circ-DONSON interacted with the NURF complex in BGC-823 cells

Through FISH assay (Fig. 3e), we found circ-DONSON was mainly localized in the nucleus. We further validated it through qRT-PCR (Fig. 4a). To further investigate the molecular mechanism, we searched the potential protein that interacts with circ-DONSON. We performed pulldown assay and silver staining. We then chose the differential band in circ-DONSON lane for mass spectrum identification. SNF2L, an essential subunit of the NURF complex, was identified (Fig. 4b). Through RIP and pulldown assay, we demonstrated their direct interaction (Fig. 4c, d). FISH assay also confirmed their colocalization in BGC-823 cells (Fig. 4e). Moreover, domain mapping assay indicated that the region 650–948 bp of circ-DONSON was essential for their interaction (Fg. 4f). EMSA assay further confirmed that SNF2L directly interacted with the region 650–948 bp of circ-DONSON (Fig. 4g). Finally, pulldown assay using circ-DONSON probes showed that circ-DONSON precipitated with SNF2L, BPTF and RBBP4 (three subunits of the NURF complex) in BGC-823 cells (Fig. 4h), indicating circ-DONSON associated with the NURF complex in GC.

### circ-DONSON recruits the NURF complex to activate SOX4 transcription

**Fig. 5:**
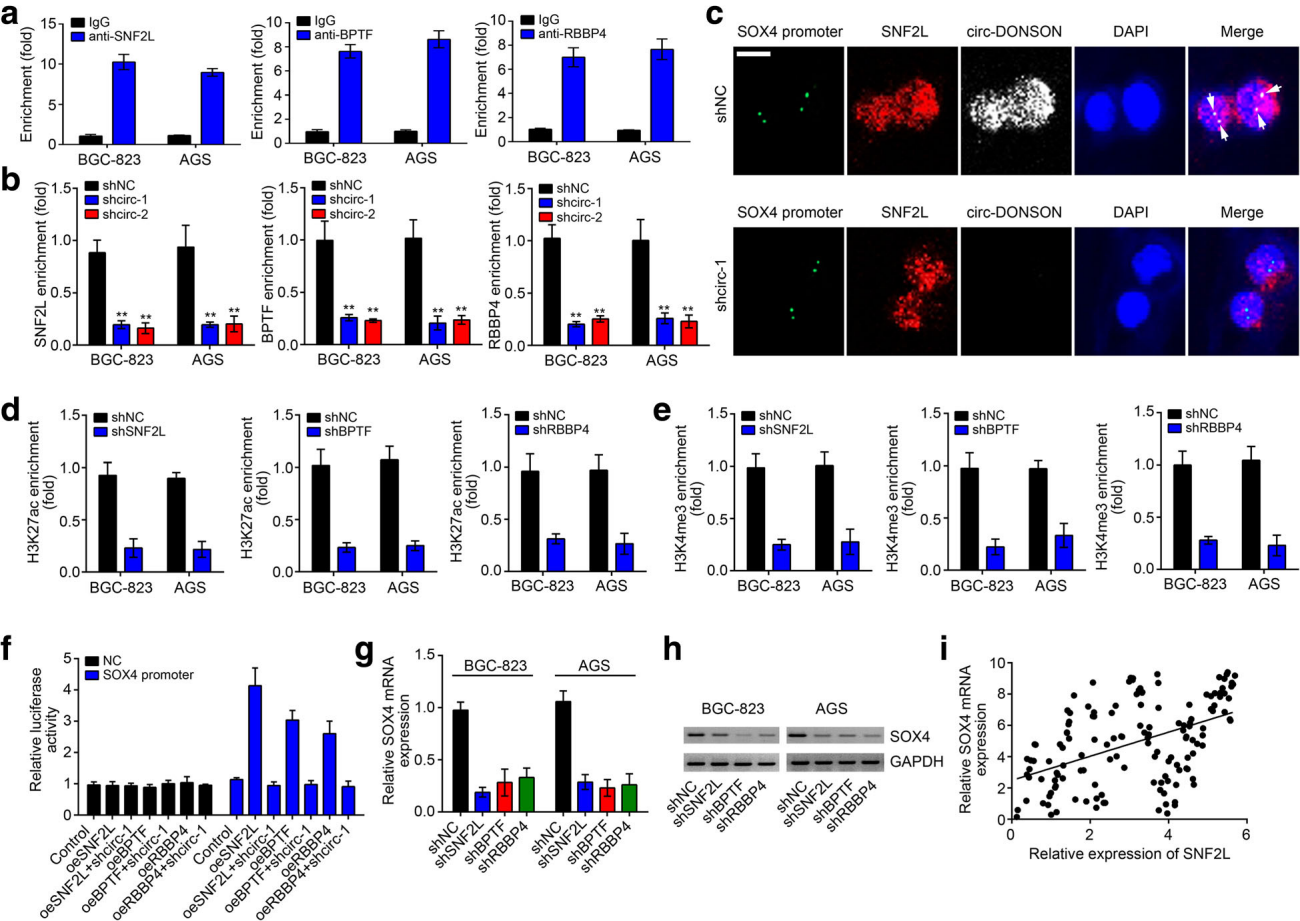
circ-DONSON recruits the NURF complex to activate SOX4 transcription. **a** ChIP assay showed that SNF2L, BPTF and RBBP4 were enriched on SOX4 promoter. **b** ChIP assay showed that circ-DONSON silencing attenuated the enrichment of SNF2L, BPTF and RBBP4 on SOX4 promoter. **c** DNA-FISH verified that circ-DONSON silencing abrogated the colocalization between SNF2L and SOX4 promoter in BGC-823 cells. Scale bar: 5 μm. **d**, **e** Depletion of SNF2L, BPTF or RBBP4 impaired the enrichment of active markers H3K27ac and H3K4me3 on SOX4 promoter. **f** Luciferase reporter assay showed that overexpression of SNF2L, BPTF or RBBP4 increased the luciferase activity while circ-DONSON silencing abrogated it. The SOX4 promoter region was constructed into the pGL3 luciferase vector. **g**, **h** qRT-PCR and western blotting analyses of SOX4 expression after knockdown of SNF2L, BPTF or RBBP4. **i** qRT-PCR analysis indicated that SOX4 expression was negatively correlated with SNF2L in GC tissues. **P < 0.01

The NURF complex is a chromatin remodeler and activates gene expression [19]. Therefore, we wondered whether the NURF complex participates in the regulation of SOX4 transcription. ChIP assay showed that SNF2L, BPTF and RBBP4 could enrich on the same region of SOX4 promoter as circ-DONSON (Fig. 5a). Notably, circ-DONSON silencing impaired the enrichment of SNF2L, BPTF and RBBP4 on SOX4 promoter (Fig. 5b). FISH assay also confirmed that circ-DONSON knockdown abrogated the colocalization of SOX4 promoter with SNF2L (Fig. 5c). Interestingly, silencing of SNF2L, BPTF or RBBP4 also suppressed the enrichment of the active markers H3K27ac and H3K4me3 on SOX4 promoter (Fig. 5d, e), indicating that the NURF complex might regulate SOX4 transcription. We further performed luciferase reporter assay and demonstrated that overexpression of SNF2L, BPTF or RBBP4 promoted the luciferase activity of SOX4 promoter while circ-DONSON silencing abrogated it (Fig. 5f), demonstrating that the NURF complex promotes SOX4 transcription in a circ-DONSON-dependent manner. Really, silencing of SNF2L, BPTF or RBBP4 decreased the expression of SOX4 in GC cells (Fig. 5g, h). Furthermore, we also observed that the expression of SNF2L was positively correlated with SOX4 in GC tissues (Fig. 5i). In summary, our data suggested that the circ-DONSON associated with the NURF complex to activate SOX4 transcription in GC.

### The NURF complex modulates GC progression

**Fig. 6:**
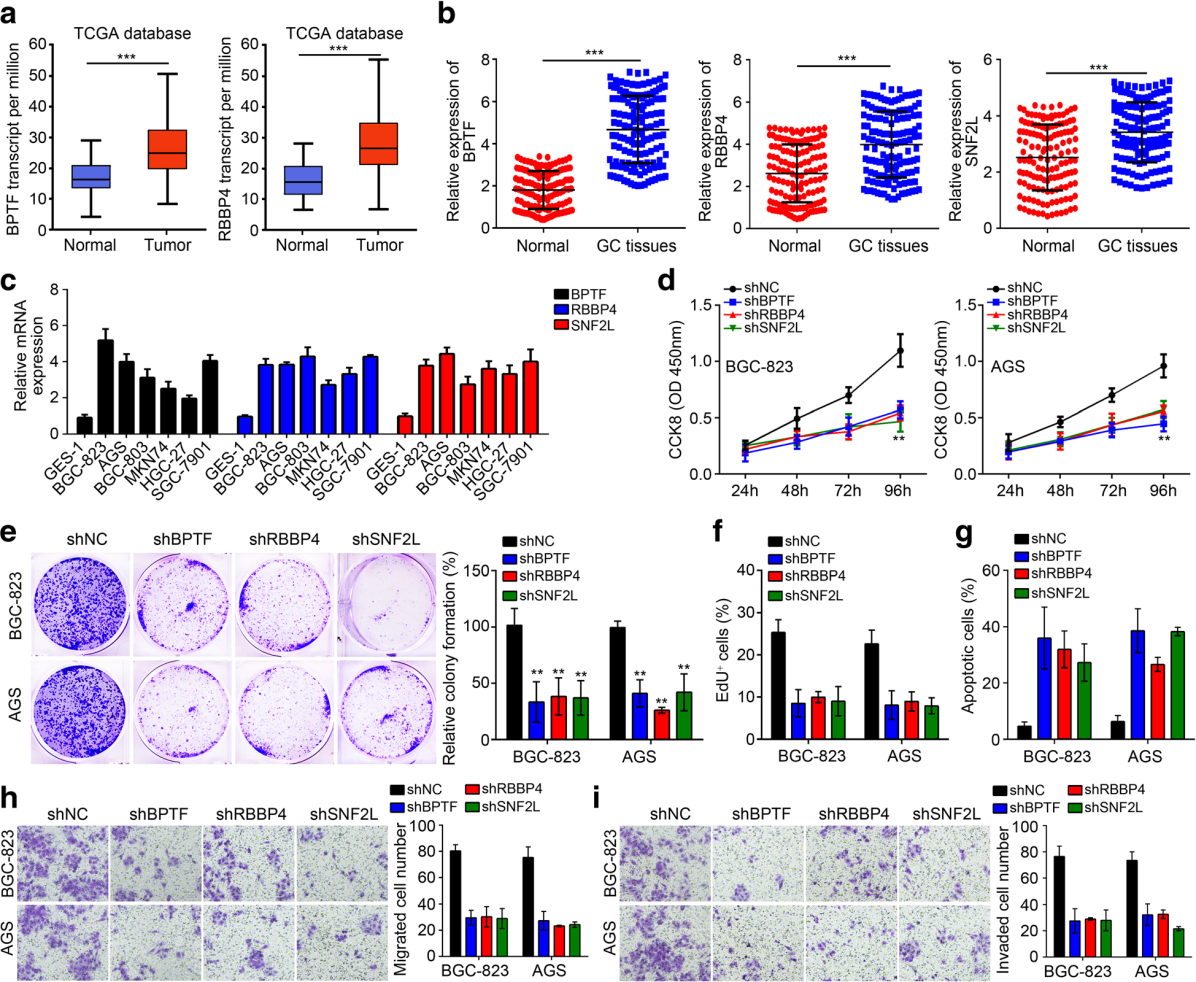
The NURF complex modulates GC progression. **a** BPTF and RBBP4 were upregulated in GC tissues according to TCGA database. **b** qRT-PCR analysis of expression levels of BPTF, RBBP4 and SNF2L in 142 GC tissues and adjacent normal tissues. **c** Relative expression of BPTF, RBBP4 and SNF2L in GC cell lines by qRT-PCR analysis. **d**-**f** CCK8, colony formation and EdU assays showed that knockdown of BPTF, RBBP4 or SNF2L suppressed proliferation of BGC-823 and AGS cells. **g** Knockdown of BPTF, RBBP4 or SNF2L promoted apoptosis of BGC-823 and AGS cells. **h**, **i** Transwell assay showed that knockdown of BPTF, RBBP4 or SNF2L inhibits migration and invasion of BGC-823 and AGS cells. **P < 0.01 and ***P < 0.001

Whether the NURF complex regulates GC progression has not been reported. Thus, we further explored the roles of the NURF complex on GC cells. Through TCGA database, we found that the expressions of BPTF and RBBP4 were significantly upregulated in GC tissues (Fig. 6a). qRT-PCR analysis also confirmed the upregulation of the NURF complex in GC tissues and cell lines (Fig. 6b, c). Then we knocked down BPTF, RBBP4 or SNF2L and performed functional experiments. CCK8, colony formation and EdU assays showed that silencing of BPTF, RBBP4 or SNF2L significantly suppressed GC cell proliferation (Fig. 6d-f). FACS analysis and Transwell assay indicated that silencing of BPTF, RBBP4 or SNF2L induced apoptosis and inhibited cell migration and invasion (Fig. 6g-i). In conclusion, these findings suggested that the NURF complex also promotes GC progression.

### Effects of circ-DONSON on GC growth in vivo

**Fig. 7:**
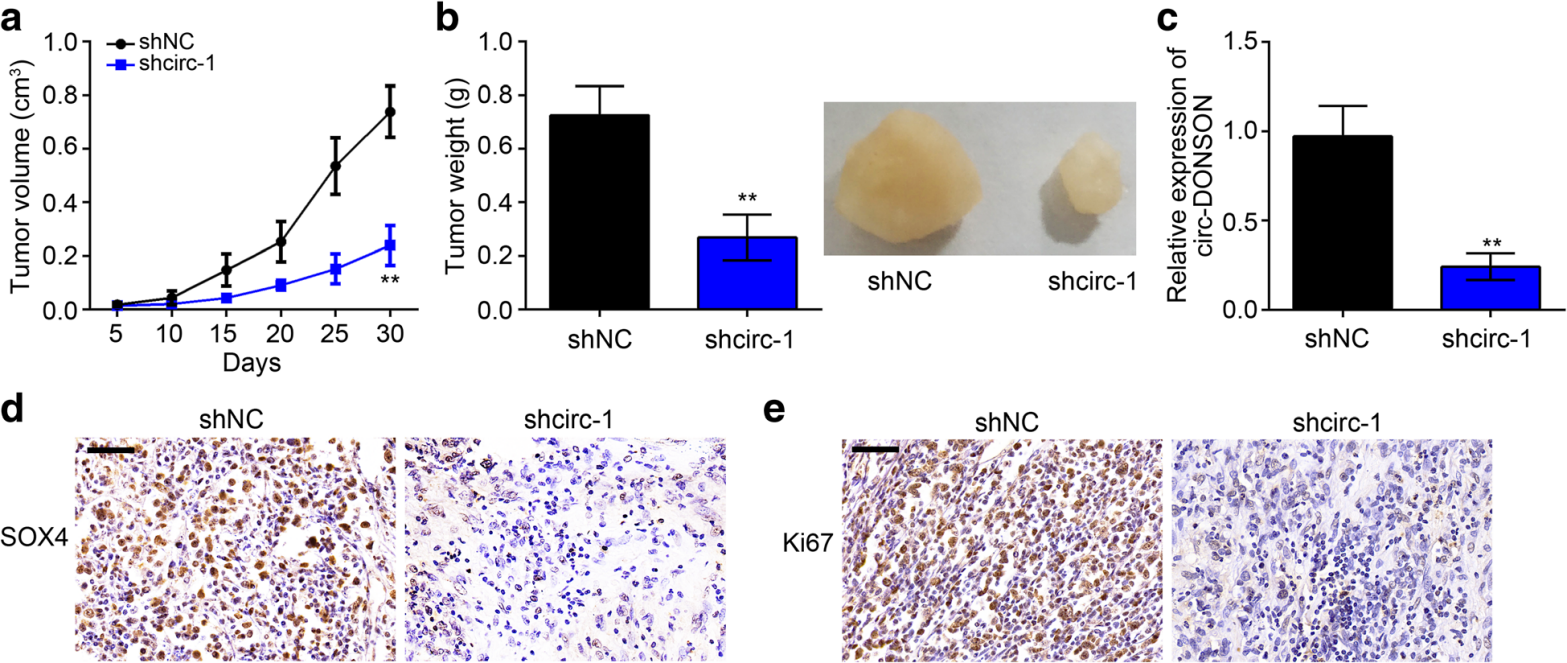
Effects of circ-DONSON on GC growth in vivo. **a** Tumor volumes were determined every 5 days. **b** 30 days after injection, the tumor weights were measured. The representative images of tumor tissues in each group were presented in the right. *n* = 4 for each group. **c** qRT-PCR analysis of circ-DONSON in tumor tissues. **d**, **e** IHC analysis of SOX4 and Ki67 expression in tumor tissues of each group. Scale bar: 50 μm. **P < 0.01

Then, we investigated the effect of circ-DONSON silencing on GC growth in vivo. BGC-823 cells with circ-DONSON knockdown or negative control were subcutaneously injected into the flank of nude mice. Every 5 days, the tumor volumes were measured and after 30 days the tumor weights were determined. Results showed that circ-DONSON knockdown significantly decreased tumor volumes and weights (Fig. 7a-c). Furthermore, immunohistochemistry for SOX4 and Ki67 was conducted to detect SOX4 and Ki67 expression. Results showed that circ-DONSON silencing led to a substantial reduce of SOX4 and Ki67 protein levels (Fig. 7d, e), indicating circ-DONSON knockdown suppresses GC growth in vivo through SOX4.

## Discussion

In this study, we investigated the functions of circ-DONSON in GC progression. circ-DONSON was highly expressed in GC tissues and cell lines. We also found that circ-DONSON overexpression predicted advanced tumor stage, metastasis and poor prognosis. Moreover, we found that circ-DONSON silencing suppressed GC cell proliferation, migration and invasion while inducing apoptosis in vitro. Animal experiments indicated that circ-DONSON knockdown suppressed GC growth in vivo. We also found that circ-DONSON associated with the NURF complex through directly interacting with SNF2L. circ-DONSON recruited the NURF complex to SOX4 promoter and initiated its transcription. Collectively, our study demonstrated that circ-DONSON is a novel oncogenic circRNA through activation of SOX4 in GC.

In the recent years, large amounts of circRNAs are identified aberrantly expressed in tumor tissues, including GC. Emerging studies showed that circRNAs are important regulators for tumorigenesis by modulating malignant behaviors of tumor cells [11, 20]. For example, circRNA hsa_circ_0000263 promotes cervical cancer progression via targeting miR-150-5p [21]. circRNA circ_0008450 is upregulated in hepatocellular carcinoma (HCC) and promotes proliferation and invasion of tumor cells [22]. In GC, only a few important circRNAs have been identified. hsa_circ_0000190 was identified as a diagnostic biomarker in GC [23]. Circular RNA_LARP4 was demonstrated to repress GC growth and invasion [5]. Additionally, Circ-SFMBT2 interacts with miR-182-5p to increase the growth of GC cells through upregulating CREB1 expression [10]. How circRNAs regulates GC development still remains ill understood. In our study, we screened a novel circRNA circ-DONSON. We demonstrated that circ-DONSON contributes to the malignant behaviors of GC cells.

To explore the molecular mechanism of circ-DONSON, we analyzed the downstream signaling pathway. We found that circ-DONSON could promote SOX4 expression in GC cells. SOX4 is a key transcription factor involved in development of several cancers. For instance, activated SOX4 signaling was reported to promote breast cancer metastasis [24]. Upregulated SOX4 by UCA1 contributes to proliferation and invasion in renal cell carcinoma [25]. SOX4 is also found to promote GC progression [26, 27]. Consistent with above evidence, we also found that increased expression by circ-DONSON regulates the proliferation, migration, invasion and apoptosis of GC cells.

Recently, most studies about circRNAs demonstrated that circRNAs could work as competing endogenous RNAs (ceRNAs) to inhibit miRNAs and play functions [10, 13]. However, in our study, we found that circ-DONSON was mainly located in the nucleus of GC cells, indicating circ-DONSON might be not a miRNA sponge. Up to date, how circRNAs exerts in the nucleus remains poorly investigated. In our study, we found that circ-DONSON directly deposited on the promoter of SOX4 and regulates its chromatin accessibility. Then through RNA pulldown and mass spectrum identification, we found that circ-DONSON interacted with SNF2L, an important subunit of the NURF complex [28]. We showed that circ-DONSON was associated with the NURF complex through directly interacting with SNF2L subunit in GC. Moreover, we demonstrated that the NURF complex was enriched on SOX4 promoter in a circ-DONSON-dependent manner. The NURF complex is a critical chromatin remodeler and regulates gene expression [19, 29, 30]. Our results also indicated that NURF depletion suppressed SOX4 transcription and decreased SOX4 mRNA levels. Thus, our research revealed that circ-DONSON/NURF axis activates SOX4 signaling in GC.

Although the NURF complex has been reported to participate in some cancers, such as intestinal tumorigenesis [31] and HCC [32], whether it is involved in GC remains undefined. In our study, we found that its subunits RBBP4, BPTF and SNF2L were highly expressed in GC tissues. And knockdown of RBBP4, BPTF or SNF2L significantly suppressed GC cell proliferation, migration and invasion and promoted apoptosis. Thus, our results illustrated that the NURF complex contributes to GC development for the first time.

## Conclusion

In summary, we identified a novel upregulated circRNA circ-DONSON that plays an oncogenic role in GC and associates with poor prognosis. Functional experiments demonstrated that circ-DONSON regulates GC cell proliferation, migration, invasion and apoptosis through the NURF complex-dependent activation of SOX4 signaling.

## Additional file

Additional file 1: **Figure S1.** circ-DONSON overexpression promotes proliferation, migration and invasion of GC cells. a Diagram of back-splicing for circ-DONSON formation. b qRT-PCR analysis of circ-DONSON expression after transfection with pcDNA3-circ-DONSON or vector control. c CCK8 assay was used for proliferation evaluation. d Colony formation assay indicated that circ-DONSON overexpression increased the colony numbers. e, f Transwell assays indicated that overexpression of circ-DONSON promoted migration and invasion of BGC-823 and AGS cells. **P < 0.01 and ***P < 0.001. **Table S1.** Sequence of circ-DONSON. (DOCX 415 kb)

## Abbreviations

ceRNA: Competing endogenous RNA
circRNA: circular RNA
FISH: Fluorescence in situ hybridization
GC: gastric cancer
IHC: Immunohistochemistry
ISH: In situ hybridization
qRT-PCR: Real-time quantitative polymerase chain reaction
RIP: RNA immunoprecipitation
