# Supplementary material for: Circular RNA circ-DONSON facilitates gastric cancer growth and invasion via NURF complex dependent activation of transcription factor SOX4
Source: Mol Cancer. 2019 Mar 28;18:45. doi: 10.1186/s12943-019-1006-2 (PMC6437893; doi:10.1186/s12943-019-1006-2)
Supplement: Supplementary file 1 — Figure S1. circ-DONSON overexpression promotes proliferation, migration and invasion of GC cells. a Diagram of back-splicing for circ-DONSON formation. b qRT-PCR analysis of circ-DONSON expression after transfection with pcDNA3-circ-DONSON or vector control. c CCK8 assay was used for proliferation evaluation. d Colony formation assay indicated that circ-DONSON overexpression increased the colony numbers. e, f Transwell assays indicated that overexpression of circ-DONSON promoted migration and invasion of BGC-823 and AGS cells. **P < 0.01 and ***P < 0.001. Table S1. Sequence of circ-DONSON. (DOCX 415 kb) [file 12943_2019_1006_MOESM1_ESM.docx]

**
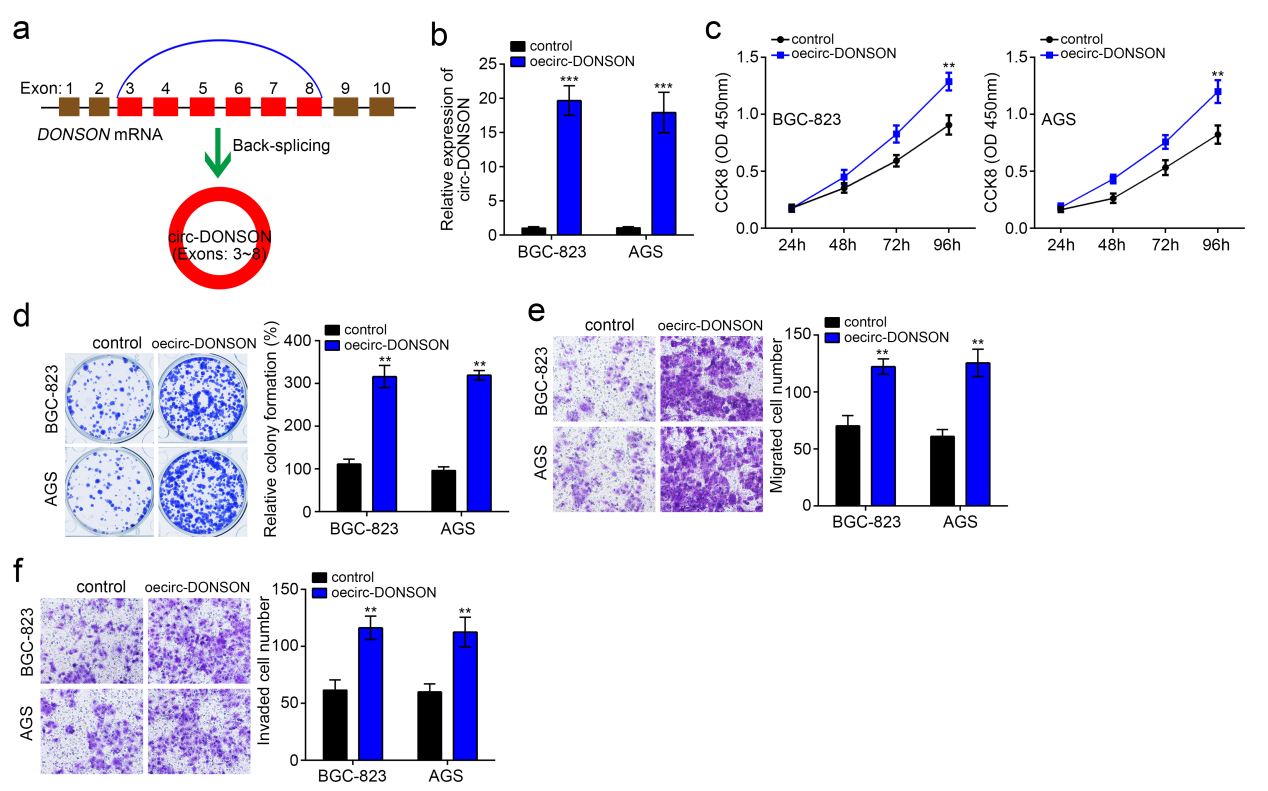
Fig. S1:** circ-DONSON overexpression promotes proliferation, migration and invasion of GC cells. **a** Diagram of back-splicing for circ-DONSON formation. **b** qRT-PCR analysis of circ-DONSON expression after transfection with pcDNA3-circ-DONSON or vector control. **c** CCK8 assay was used for proliferation evaluation. **d** Colony formation assay indicated that circ-DONSON overexpression increased the colony numbers. **e, f** Transwell assays indicated that overexpression of circ-DONSON promoted migration and invasion of BGC-823 and AGS cells. ***P*<0.01 and ****P*<0.001.

**table S1:** Sequence of circ-DONSON.

| ACUUCACAUGUAUCAUUCUCCGAGCCUGAUAUUCCGUCCUCAAAAAGUACUGAGUUACCUGUGGACUGGAGUAUUAAAACGCGACUCCUUUUCACCUCUUCUCAACCCUUUACCUGGGCAGAUCAUUUGAAAGCACAGGAAGAAGCUCAAGGUCUUGUCCAGCAUUGUAGGGCAACAGAAGUUACUUUGCCUAAAAGUAUACAGGAUCCCAAACUCUCCUCUGAGCUCCGUUGUACCUUCCAGCAGAGCCUUAUCUAUUGGCUCCACCCUGCUUUGUCUUGGCUACCACUGUUCCCUCGUAUUGGAGCUGAUAGAAAAAUGGCUGGAAAGACAAGUCCUUGGUCAAAUGAUGCAACCCUGCAGCAUGUUUUAAUGAGUGACUGGUCUGUGAGCUUUACUUCUCUAUAUAAUUUGCUGAAGACAAAACUUUGCCCCUAUUUCUACGUUUGUACCUAUCAGUUUACUGUCCUGUUCCGAGCAGCAGGAUUAGCUGGAAGUGACUUAAUCACAGCUCUCAUAUCUCCAACAACUCGAGGUUUAAGAGAAGCUAUGAGAAAUGAAGGUAUUGAAUUUUCUCUGCCUUUAAUAAAAGAAAGUGGCCAUAAGAAGGAGACAGCAUCUGGAACAAGCUUGGGAUAUGGGGAGGAGCAAGCCAUCAGUGAUGAGGAUGAAGAGGAAAGUUUUUCCUGGCUGGAAGAGAUGGGUGUGCAAGAUAAAAUUAAAAAGCCAGACAUACUUUCUAUCAAGCUGCGUAAAGAGAAACAUGAAGUACAAAUGGAUCACAGACCUGAAUCUGUUGUGUUGGUAAAAGGAAUCAACACCUUUACAUUGCUCAAUUUUUUGAUUAACUCUAAGAGUUUAGUUGCUACCUCAGGUCCACAGGCAGGACUUCCUCCAACCCUCUUGUCCCCUGUUGCUUUCCGAGGUGCCACAAUGCAAAUGCUUAAG |
| --- |
